# Dose-dependent anxiolytic and antidepressant-like effects of chronic oxytocin in corticosterone-induced female mouse model of anxiety and depression

**DOI:** 10.1016/j.bbrep.2026.102510

**Published:** 2026-02-18

**Authors:** Masayoshi Mori, Misaki Tamura, Norihiro Sumi, Hiroyoshi Harada, Yusuke Murata

**Affiliations:** Department of Pharmacotherapeutics, Faculty of Pharmaceutical Sciences, Fukuoka University, Fukuoka, 8-19-1, Nanakuma, Jonan-ku, Fukuoka, 814-0180, Japan

**Keywords:** Oxytocin, Female, Depression, Anxiety, Dose, Corticosterone

## Abstract

Oxytocin (OXT) has therapeutic effects on psychiatric disorders, such as anxiety and depression, in both animals and humans; however, an increasing number of OXT treatment studies have reported conflicting results. Although the effects of OXT on emotion regulation vary depending on factors such as sex and dosage, the dose-dependent effects of chronic OXT administration remain unclear, particularly in women. In this study, we aimed to assess the dose-dependent effects of chronic OXT administration on emotional behavior in female mice with corticosterone (CORT)-induced anxiety and depression. A total of 58 female C57BL/6J mice received daily co-administration of OXT (0.1 or 1 mg/kg, intraperitoneal) and/or CORT (40 mg/kg, subcutaneous) for 4 weeks, and their anxiety- (open field and elevated plus maze tests) and depression-like behaviors (forced swimming and tail suspension tests) were evaluated. A 0.1 mg/kg dose of OXT blocked the CORT-induced increase in anxiety-like behavior (open field test) and depression-like behavior (forced swimming test), whereas the 1 mg/kg dose did not. Similarly, a dose-dependent effect of OXT was observed in the elevated plus maze and tail suspension tests. Furthermore, the 1 mg/kg dose of OXT significantly increased plasma OXT levels. These findings suggest that a certain level of OXT signaling activity is needed to exert anxiolytic and antidepressant-like effects, which may lead to a non-linear dose-dependent effect of OXT in a female mouse model of CORT-induced anxiety and depression. Targeting dose-dependent OXT signaling is a potential therapeutic strategy for women with psychiatric disorders.

## Highlights

• Simultaneous oxytocin (0.1 mg/kg) blocked the aversive effects of corticosterone.
• Simultaneous oxytocin (1 mg/kg) exerted any effects on emotional behaviors.
• Simultaneous oxytocin (1 mg/kg) elevated peripheral oxytocin levels.
• Oxytocin regulated emotion in a non-linear dose-dependent manner in female mice.

## 1 Introduction

Depression and anxiety are highly prevalent and disabling psychiatric disorders [1,2]. Although several antidepressants are available, only approximately half of the treated patients achieve remission. This limitation is partly due to the incomplete understanding of the molecular mechanisms underlying these disorders [[3], [4], [5]]. Therefore, identifying novel pharmacological targets that regulate core psychiatric symptoms is a critical research priority.

Oxytocin (OXT), a neuropeptide primarily synthesized in the paraventricular and supraoptic nuclei of the hypothalamus, is released both centrally and peripherally in response to stimuli such as social interaction, sexual activity, and suckling [6]. OXT-producing neurons project to brain regions involved in emotional and stress regulation [7], suggesting a close relationship between OXT signaling and emotional behavior. OXT has been shown to modulate neural function in these regions, produce anxiolytic effects in humans, and buffer stress responses, supporting its therapeutic potential for psychiatric disorders [[8], [9], [10], [11]].

While increasing preclinical and clinical evidence supports the antidepressant- and anxiolytic-like properties of OXT, a growing number of studies have reported inconsistent or conflicting outcomes [12]. Most studies on acute OXT administration report beneficial behavioral effects [[13], [14], [15], [16], [17]], but findings on chronic OXT administration remain conflicting. For instance, OXT treatment has been shown to exert anxiolytic-like effects [18,19], neutral effects [20,21], or even anxiogenic-like outcomes [22]. Importantly, the effects of OXT on emotion regulation differ depending on sex [12,[23], [24], [25]]. Acute intranasal OXT treatment reduced the activity of amygdala, a key brain region involved in the perception of fearful and threatening stimuli, in response to negative faces and threatening scenes in healthy men [26]. In contrast, healthy women administered OXT exhibited greater amygdala responses when viewing threatening scenes [27]. In addition, chronic intraperitoneal OXT injection increased social behavior in adult male rats, but no effect was observed in adult female rats [28]. These studies suggest that various factors, such as the dosage administered and sex, are crucial to determine the effects of OXT on emotion regulation.

The lifetime risk of depression is approximately 21% for women and 13% for men [29]. In addition, women with depression tend to have more severe symptoms compared with men [30]. Although the detailed mechanisms underlying the sex disparity in psychiatric disorders remain to be elucidated, sex hormones are thought to drive sex differences in the risk of anxiety disorders and depression [[31], [32], [33], [34]]. Importantly, OXT has been reported to interact with ovarian hormones, such as estrogen [28,35]. However, to date, most studies on the anxiolytic- and antidepressant-like effects of OXT have focused on males, acute dosing regimens, and single-dose designs [12,36], which limit their translational relevance. Given that psychiatric conditions, including depression, typically require long-term treatment, the therapeutic effects of chronic OXT administration on emotion regulation in females should be examined. Chronic OXT treatment was reported to alleviate depression-like behavior in a female rodent model of depression [18,37], but these results were obtained using a single-dose of OXT. As such, further research is warranted to understand the dose-dependent effects of chronic OXT administration in females with psychiatric conditions.

Hypothalamic-pituitary-adrenal (HPA) axis is one of the endocrinological systems that regulate stress responses [38], and its dysregulation causes psychiatric disorders, including depression [39]. Chronic administration of exogenous corticosterone (CORT) reflects chronic stress conditions and can mimic dysregulation of the HPA axis in rodents. This CORT model produces not only anxiety- and depression-like behaviors but also physiological changes commonly observed in human depression [40]. Of note, many of the behavioral and physiological changes associated with chronic CORT administration can be reversed with antidepressant treatment [41,42], supporting the predictive validity of CORT administration in inducing depression in animals.

Chronic administration of glucocorticoids, including CORT, is primarily achieved through drinking water or subcutaneous injection [43]. Although each administration method has distinct advantages and disadvantages, such as degree of administration stress and dosage control, both methods are feasible and appropriate for inducing an animal model of depression [40,42,44,45]. We previously used chronic subcutaneous exposure to dexamethasone, a synthetic glucocorticoid, to induce an anxiety and depression phenotype [18]. Based on these observations, in this study, we investigated the therapeutic effects of chronic OXT administration in female mouse models of anxiety and depression induced by chronic exposure to subcutaneous CORT. We then evaluated the anxiolytic- and antidepressant-like effects of two OXT doses (0.1 and 1 mg/kg body weight) to explore the dose–response relationship in a female-specific context.

## 2 Materials and methods

### 2.1 Animals

Fifty-eight female C57BL/6J mice (8 weeks old at arrival; 16–23 g; Jackson Laboratory Japan, Inc., Kanagawa, Japan) were used in this study. Mice were individually housed under a 12:12-h light–dark cycle, with lights on at 07:00 a.m., and had free access to food and water. All procedures were approved by the University of Fukuoka Committee on Animal Research (January 14, 2022; approval number: 2113103).

### 2.2 Drug administration

OXT (4016373, Bachem, Torrance, CA, USA) was dissolved in saline. CORT (C2505, Sigma-Aldrich, St. Louis, MO, USA) was dissolved in saline containing 15% Tween 80 and 2% dimethyl sulfoxide. Mice were subcutaneously injected with 40 mg/kg of CORT, as this dose has been demonstrated to reliably increase anxiety- and depression-like behaviors without altering nonspecific motor activity [44,45]. Mice were intraperitoneally administered OXT at either 0.1 mg/kg (low dose) or 1 mg/kg (high dose) and subcutaneously injected with CORT 30 min later. The doses of OXT and drug administration protocols were based on previously described methods with minor modifications [9]. All injections were administered at 0.1 mL/10 g body weight. Injections were given once daily between 11:30 a.m. and 1:00 p.m. for 28 consecutive days. To minimize and equalize the non-specific stress effects of daily handling and injection on mice, the drug injection processes in all groups were performed by a well-trained experimenter.

### 2.3 Experimental design

**Fig. 1:**
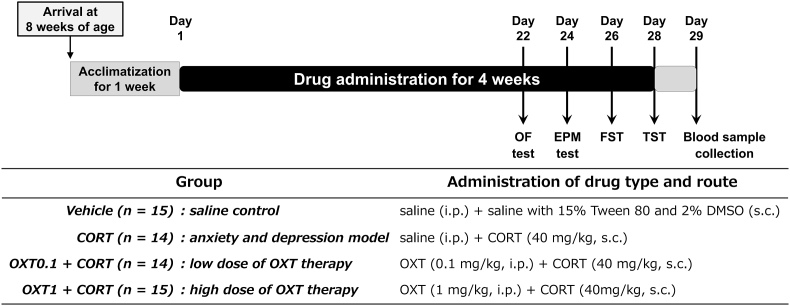
**Experimental design.** All mice received either the vehicle for OXT (saline, i.p.), the vehicle for CORT (saline with 15% Tween 80 and 2% DMSO, s.c.), CORT (40 mg/kg, s.c.), or OXT (0.1 or 1 mg/kg, i.p.) + CORT (40 mg/kg, s.c.) once daily for 4 weeks. Beginning 3 weeks after treatment initiation, mice were sequentially subjected to a battery of behavioral tests (open field, elevated plus maze, forced swimming, and tail suspension tests) on alternate days. Mice were euthanized by decapitation 24 h after the TST for evaluation of plasma OXT levels. OXT: oxytocin; CORT: corticosterone; i.p.: intraperitoneal injection; s.c.: subcutaneous injection; DMSO: dimethyl sulfoxide.

After a 1-week acclimation, mice were randomly assigned to four groups: (1) vehicle (n = 15), injected with vehicles for OXT and CORT; (2) CORT (n = 14), saline and CORT (40 mg/kg); (3) OXT0.1 + CORT (n = 14), OXT (0.1 mg/kg) and CORT (40 mg/kg); and (4) OXT1 + CORT (n = 15), OXT (1 mg/kg) and CORT (40 mg/kg). Beginning on day 22, mice underwent a battery of behavioral tests on alternate days. All tests were conducted between 9:30 a.m. and 11:30 a.m. under white light, with experimenters blinded to treatment. Mice were euthanized under anesthesia 24 h after the final behavioral test, and trunk blood was collected for plasma analysis (see Fig. 1).

### 2.4 Open field (OF) test

The OF test was performed in a circular arena (diameter: 60 cm; wall height: 50 cm). Mice were placed in the center and allowed to explore for 5 min. Behavior was recorded via an overhead camera. Fewer entries into the central zone indicated increased anxiety-like behavior. Locomotor activity, measured as total distance traveled, was analyzed using video-tracking software (SMART, Panlab Harvard Apparatus, Barcelona, Spain).

### 2.5 Elevated plus maze (EPM) test

The EPM test consisted of two closed arms (6 × 31.5 cm; wall height: 15 cm) and two open arms of the same dimensions, elevated 40 cm above the floor. The arms extended from a central platform (6 × 6 cm). Mice were placed in the center facing an open arm and allowed to explore for 5 min. Fewer entries into and reduced time spent in open arms were interpreted as anxiety-like behavior. Total arm entries were used as an index of locomotor activity.

### 2.6 Forced swimming test (FST)

Each mouse was placed in a glass cylinder (diameter: 14 cm; height: 20 cm) filled with water to a depth of 12 cm (25 ± 1 °C) and forced to swim for 6 min. The duration of immobility during the final 4 min was recorded. Longer immobility times indicated depression-like behavior. The water was changed and the cylinder cleaned after each trial.

### 2.7 Tail suspension test (TST)

Mice were suspended by the tail for 6 min using a clip apparatus (MSBOX2007, YTS Yamashita Giken, Tokushima, Japan). Immobility time was recorded during the last 4 min. Mice were considered immobile only when hanging passively and motionless. Longer durations of immobility reflected depression-like behavior.

### 2.8 Plasma OXT level

Trunk blood was collected in tubes containing 5 μL heparin. Samples were centrifuged at 1000×*g* for 15 min at 4 °C, and the plasma was stored at −80 °C. OXT concentrations were quantified using a commercial OXT ELISA kit (500440, Cayman Chemical, Ann Arbor, MI, USA) according to the manufacturer's instructions.

### 2.9 Statistical analysis

Data were analyzed using R software (version 4.3.1; R Development Core Team, Vienna, Austria). All data were compared among the four groups (Vehicle, CORT, OXT0.1 + CORT, and OXT1 + CORT). Normality was assessed via the Shapiro–Wilk test; homogeneity of variance was tested using Levene's test. One-way analysis of variance (ANOVA), followed by Shaffer's modified sequentially rejective Bonferroni procedure was used for normally distributed data. Nonparametric data—including closed-arm entries in the EPM test, immobility duration in the FST, and plasma OXT levels—were analyzed using the Kruskal–Wallis test followed by Steel–Dwass post hoc comparisons. Results are expressed as mean ± standard error of the mean. Statistical significance was set at *P* < 0.05.

## 3 Results

### 3.1 Effects of chronic OXT and CORT treatment on anxiety-like behavior

**Fig. 2:**
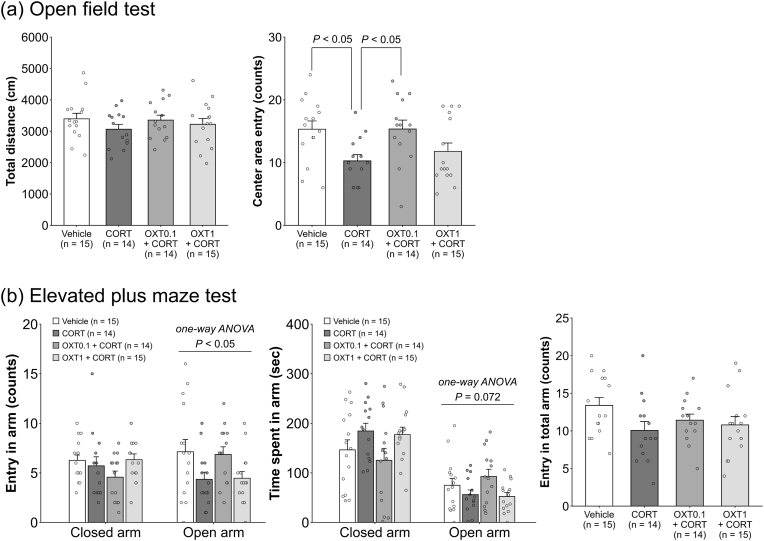
**Dose-dependent effects of chronic OXT and CORT administration on anxiety-like behaviors in female mice.** Chronic OXT and CORT administration did not affect locomotor activity in the OF (a, *left*) and EPM tests (b, *right*). Chronic CORT significantly reduced center entries in the OF test, an effect reversed by co-treatment with 0.1 mg/kg OXT (a, right). Drug treatment significantly influenced open arm entries in the EPM test among the four groups; however, post hoc comparisons did not reveal significant pairwise differences (b, left). Group size: 14–15 animals. Bars represent mean ± standard error of the mean. Statistical tests: Kruskal–Wallis test with Steel–Dwass post hoc test (OF test center entries); one-way ANOVA with Shaffer's modified sequentially rejective Bonferroni post hoc test (total distance in OF and EPM tests). OXT: oxytocin; CORT: corticosterone; EPM: elevated plus maze; OF: open field.

Fig. 2 shows the effects of chronic CORT and OXT administration on anxiety-like behaviors. In the OF test, no significant differences were observed in total distance traveled among the four groups (Fig. 2a, left), indicating that locomotor activity was not affected by treatment. The Kruskal–Wallis test revealed significant differences in the number of central area entries (H = 9.990, *P* = 0.018; Fig. 2a, right). Post hoc analysis showed that the CORT group exhibited fewer center entries compared to both the vehicle and OXT0.1 + CORT groups (*P* < 0.05 for both), suggesting increased anxiety-like behavior induced by CORT and a reversal by low-dose OXT. In the EPM test, the Kruskal–Wallis test indicated no significant group differences in the number of closed-arm entries among the four groups (Fig. 2b, left of left panel). Similarly, no significant change in time spent in the closed arm was observed among the four groups (Fig. 2b, left of middle panel). Although one-way ANOVA of the time spent in the open arm did not reach statistical significance, we observed a tendency toward a difference in the time spent in the open arm among the four groups (F_(3,54)_ = 2.470, *P* = 0.072; Fig. 2b, right of middle panel). One-way ANOVA revealed a significant group difference in open-arm entries (F_(3,54)_ = 2.832, *P* = 0.047; Fig. 2b, right of left panel); however, post-hoc comparisons did not yield statistically significant differences between any specific groups. Total arm entries in the EPM test did not differ significantly among groups, further supporting that overall activity levels were unaffected by treatment.

### 3.2 Effects of chronic OXT and CORT treatment on depression-like behavior and plasma OXT level

**Fig. 3:**
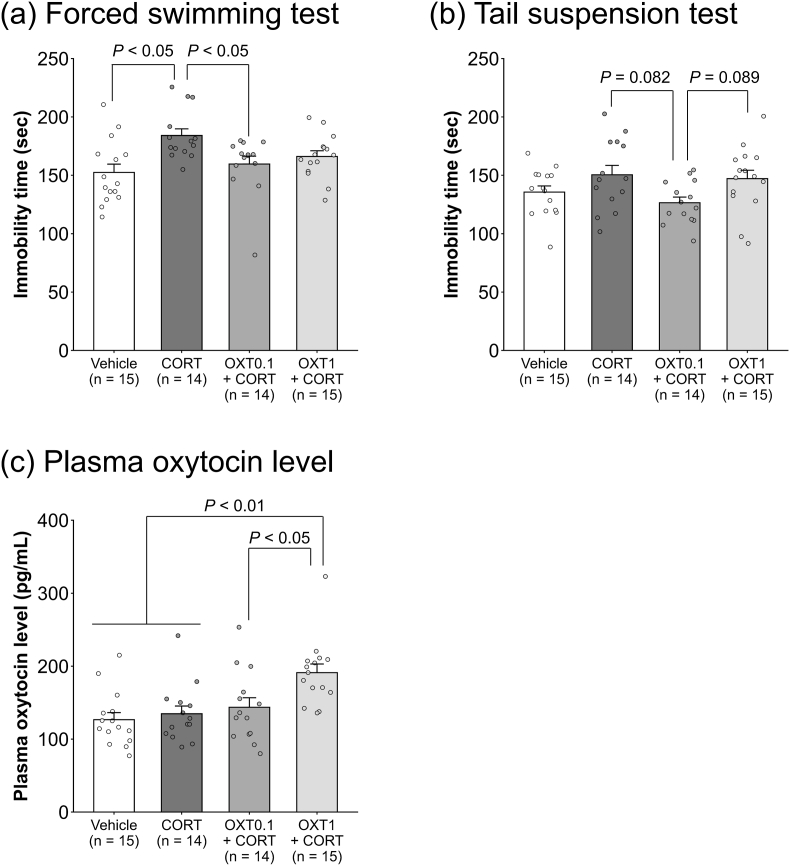
**Dose-dependent effects of chronic OXT and CORT administration on depression-like behaviors and plasma OXT level in female mice.** Chronic CORT significantly increased immobility duration in the FST, an effect reversed by 0.1 mg/kg OXT co-treatment (a). In the TST, 0.1 mg/kg OXT tended to reduce immobility duration relative to both the CORT group and the 1 mg/kg OXT co-treatment group, though differences did not reach statistical significance (b). Chronic co-treatment with 1 mg/kg OXT significantly increased plasma OXT levels (c). Group size: 14–15 animals. Bars represent mean ± standard error of the mean. Statistical tests: Kruskal–Wallis test with Steel–Dwass post hoc test (FST immobility and plasma OXT); one-way ANOVA with Shaffer's modified sequentially rejective Bonferroni post hoc test (TST immobility). OXT: oxytocin; CORT: corticosterone; FST: forced swimming test; TST: tail suspension test.

Fig. 3 summarizes the effects of OXT and CORT treatment on depression-like behaviors and plasma OXT levels. In the FST, the Kruskal–Wallis test indicated a significant group difference in immobility duration (H = 12.036, *P* = 0.007; Fig. 3a). Post hoc analysis showed significantly longer immobility in the CORT group than in the vehicle and OXT0.1 + CORT groups (*P* < 0.05 for both), indicating a depressive-like phenotype reversed by low-dose OXT. In the TST, one-way ANOVA revealed significant group differences (F_(3,54)_ = 2.777, *P* = 0.049; Fig. 3b). Although post hoc analysis did not reach statistical significance, a trend toward reduced immobility was observed in the OXT0.1 + CORT group relative to the CORT (*P* = 0.082) and OXT1 + CORT groups (*P* = 0.089), suggesting a possible antidepressant-like effect of low-dose OXT. The Kruskal–Wallis test showed a significant difference in plasma OXT levels across groups (H = 16.749, *P* = 0.008; Fig. 3c). Post hoc analysis revealed significantly higher plasma OXT levels in the OXT1 + CORT group compared to the vehicle (*P* < 0.01), CORT (*P* < 0.01), and OXT0.1 + CORT groups (*P* < 0.05), confirming successful dose-dependent elevation of peripheral OXT.

## 4 Discussion

Using two doses of OXT (0.1 and 1 mg/kg/day), we investigated the therapeutic effects of daily peripheral OXT administration for 28 days in female mice with CORT-induced depression and anxiety. CORT exposure increased anxiety- and depression-like behaviors, whereas simultaneous low-dose (0.1 mg/kg) chronic OXT treatment blocked these adverse effects, but high-dose (1 mg/kg) OXT did not. These findings suggest that chronic OXT treatment may not exert anxiolytic or antidepressant-like effects in a linear dose-dependent manner in this female mouse model.

Both the OF and EPM tests are widely used to assess anxiety-like behaviors, measuring unconditioned avoidance of aversive stimuli such as the center zone in a novel environment (OF test) or elevated open arms (EPM test) [46]. Increased immobility in the FST and TST is commonly used as a behavioral indicator of depression-like symptoms in rodents [47,48]. In this study, chronic CORT exposure elevated anxiety- and depression-like behaviors in the OF test and FST. Although both the Kruskal–Wallis test and one-way ANOVA showed significant group effects in the EPM test and TST, we did not observe significant differences between the vehicle and CORT groups in those tests. Notably, low-dose OXT treatment counteracted CORT-induced behavioral changes without affecting locomotor activity. These findings are consistent with previous reports showing that chronic CORT administration increases emotional disturbances in rodents [19,49], and that OXT plays a modulatory role in anxiety and depression [11,50]. Our results align with prior work demonstrating anxiolytic and antidepressant-like effects of OXT in animal models of psychiatric disorders [18,19,51], suggesting that chronic OXT administration may improve stress-induced behavioral impairments in females.

Importantly, although low-dose OXT reversed the effects of CORT, high-dose OXT failed to produce similar therapeutic benefits. These findings suggest that OXT's therapeutic effects on emotional behavior may follow a non-linear dose-dependent pattern. This interpretation aligns with reports showing that OXT exhibits inverted-U dose–response functions [22,23,52,53]. For instance, Peters et al. [22] reported that chronic intracerebroventricular infusion of low-dose OXT (1 ng/h) reduced anxiety in non-stressed male mice, whereas a higher dose (10 ng/h) induced anxiogenic effects. Likewise, OXT modulates social behavior in an inverted-U manner [23]. Our findings thus support the hypothesis that OXT's efficacy depends on its proximity to the peak of this inverted U-shaped dose-response curve: the low dose may reside near the optimal therapeutic window, whereas the high dose may exceed it, diminishing efficacy. To validate our hypothesis and further understand the relationship between OXT and emotion regulation, future studies should assess the dose-dependent effects of OXT on emotion regulation using an intermediate dose (0.5 mg/kg) and a broader dose range (<0.1 mg/kg and >1 mg/kg doses of OXT).

In this study, plasma OXT levels were significantly elevated in only the high-dose OXT group, but the OXT levels of the other groups were similar. However, the high-dose OXT did not show any effect on anxiety and depression, suggesting that peripheral OXT systems are not directly involved in emotion regulation in a non-linear dose-dependent manner. Although the precise mechanisms underlying OXT's non-linear effects remain unclear, several studies suggested that changes in the central OXT system activity, depending on OXT levels in the brain, are crucial for emotion regulation [22,23,54,55]. The correlation between peripheral and central OXT levels remains controversial [56,57]. Some studies suggest that peripheral OXT levels may correlate with central concentrations [[58], [59], [60]], but another report described weak correlations between plasma and central OXT levels [61]. Based on these results, we speculate that changes in the central OXT levels according to the administered OXT dosage, rather than peripheral OXT levels, were involved in the behavioral changes observed in this study. For example, Peters et al. [22] found that high-dose, but not low-dose, OXT reduced OXT receptor binding in the median raphe nucleus involved in anxiety regulation. Liu et al. [54] reported that increased OXT levels and upregulated expression of OXT receptors in the hippocampus and prefrontal cortex, key treatment regions for neurodevelopmental disorders, impaired the emotions of a female rat model of autism. Further, OXT modulates social reward and activity in the mesolimbic regions in an inverted-U dose-dependent manner [23]. Since reward circuits are essential for emotion regulation, alterations in their function contribute to psychiatric symptoms such as depression [62]. Therefore, these reports suggest that chronic OXT administration alters the expression of OXT receptors or signaling activity of brain regions associated with emotion regulation in an inverted-U dose-dependent manner. Based on these findings, we speculate that CORT alone may decrease OXT levels in the brain, 0.1 mg/kg OXT may improve OXT levels to an optimal therapeutic range in the brain, and 1 mg/kg may exceed the central system's saturation threshold, reducing its efficacy. We acknowledge the lack of mechanistic experiments as a major limitation of this study. Therefore, future studies should examine changes in the OXT system, including OXT levels, OXT receptor binding, and downstream signaling cascades, in the brain regions to better understand the effects of OXT.

In addition to this limitation, we also did not assess the dose-dependent effects of OXT alone (in the absence of CORT) on emotional behavior. Previous studies demonstrated that high-dose OXT (0.25–1 mg/kg) has intrinsic adverse effects, such as sedation, that would affect behavioral activity [28,63]. Therefore, the sedative effect of high-dose OXT (1 mg/kg) may have induced behavioral changes in this study. However, the sedative effect of OXT is observed within 5–10 min after injection [63]. In this study, OXT administration was performed after each behavioral test, with an interval of approximately 24 h between OXT administration and behavioral test. Additionally, we observed no significant dose-dependent effects of OXT on the total distance traveled in the OF test. Similarly, the dosage of OXT did not affect total arm entries in the EPM test. These results support the fact that behavioral activity levels were unaffected by OXT treatment. Therefore, we presume that high-dose OXT has low potential for sedative effects. However, the present study results should be interpreted with caution. Some studies have reported that OXT interacts with glucocorticoids, such as CORT. For example, stress-induced release of OXT within the brain and periphery are both modulated by CORT [64]. Likewise, CORT implants increased OXT binding to OXT receptors in the hippocampus [65]. OXT receptor is a member of the G-protein coupled receptor superfamily and can be coupled to Gq and Gi/o, activating or inhibiting intracellular signaling cascade [66]. The cAMP response element-binding protein (CREB) and its transcriptional targets, such as brain-derived neurotrophic factor (BDNF), play crucial roles in regulating neuronal growth and neuroplasticity of various brain regions, including the hippocampus [67,68]. Furthermore, hippocampal CREB-BDNF signaling activity contributes to anxiety and depressive symptoms [[69], [70], [71]]. Since CORT and OXT receptor play a significant role in modulating the CREB-BDNF signaling activity of the hippocampus [18,19], simultaneous CORT administration may have resulted in divergent dose-dependent effects of OXT on emotional behaviors via the OXT receptor coupled to Gq or Gi/o signaling. Therefore, to confirm the effects of OXT and make a valid conclusion, future studies should investigate the molecular mechanisms underlying the divergent effects of chronic OXT administration on emotion regulation at varying doses. Finally, the exclusive use of female mice is a limitation of this study. Since the effects of OXT on emotion regulation differ by sex [12,[23], [24], [25]], current findings cannot be directly extrapolated to males. Future studies should directly compare the dose-dependent effects of chronic OXT administration on emotional behaviors between males and females.

## 5 Conclusions

In summary, chronic CORT exposure increased anxiety- and depression-like behaviors. Chronic OXT treatment at a low dose (0.1 mg/kg) blocked the adverse effects of CORT; however, high-dose (1 mg/kg) OXT showed no effect on emotion regulation in the female mice. To our knowledge, this study is the first to demonstrate that chronic OXT treatment exerts both antidepressant- and anxiolytic-like effects in a non-linear dose-dependent manner in female mice models of CORT-induced depression and anxiety. In addition, blood OXT levels were elevated by only chronic high-dose OXT administration. These results suggest that OXT concentration in the brain regions associated with emotion regulation may vary depending on the dosage of OXT, leading to divergent effects on emotion regulation in a non-linear dose-dependent manner. Future studies are needed to elucidate the mechanisms underlying the varying effects of chronic OXT administration on emotion regulation. Although OXT has shown promise as a pharmacotherapy for psychiatric disorders, an increasing number of studies have reported conflicting findings. The present study showed that OXT dosage is a critical factor that determines the therapeutic effects of OXT on emotion regulation and may account for the variability in treatment outcomes. Our findings underscore the importance of identifying optimal dosing strategies for OXT as a potential therapeutic agent for managing depression and anxiety disorders in women.

## Author contributions CRediT

Conceptualization: Masayoshi Mori.

Data curation: Masayoshi Mori, Misaki Tamura, Hiroyoshi Harada.

Formal analysis: Masayoshi Mori, Misaki Tamura, Norihiro Sumi, Hiroyoshi Harada.

Funding acquisition: Masayoshi Mori, Yusuke Murata.

Investigation: Masayoshi Mori, Misaki Tamura, Norihiro Sumi.

Methodology: Masayoshi Mori, Yusuke Murata.

Project administration: Masayoshi Mori.

Supervision: Masayoshi Mori.

Validation: Masayoshi Mori, Misaki Tamura, Norihiro Sumi.

Visualization: Masayoshi Mori, Hiroyoshi Harada.

Writing-original draft: Masayoshi Mori, Misaki Tamura, Norihiro Sumi.

Writing-review and editing: Masayoshi Mori, Hiroyoshi Harada, Yusuke Murata.

## Declaration of generative AI and AI-assisted technologies in the writing process

The authors declare nonuse of generative AI and AI-assisted technologies in the writing process.

## Funding

This work was supported in part by the 10.13039/501100001691Japan Society for the Promotion of Science 10.13039/501100001691KAKENHI (Grant No. 23K07000).

## Declaration of competing interest

The authors declare no competing interests.

## Data Availability

Data will be made available on request.

## Glossary

**Anxiolytic**: A substance or intervention that reduces symptoms of anxiety
**Antidepressant-like effect**: A behavioral outcome in animal models that resembles the therapeutic effects of antidepressants in humans, typically measured using validated tests such as the forced swimming test or tail suspension test
**Corticosterone (CORT)**: A steroid hormone used in animal research to model stress-related disorders; chronic administration induces anxiety- and depression-like behaviors in rodents
**Forced Swimming Test (FST)**: A behavioral assay used to assess depressive-like behavior in rodents, based on the duration of immobility when placed in water
**Open Field (OF) Test**: A common test used to evaluate anxiety-like behavior and general locomotor activity in rodents by monitoring their movement in an open arena
**Elevated Plus Maze (EPM) Test**: An experimental setup for measuring anxiety-like behavior in rodents, based on their preference for open or closed arms of a plus-shaped elevated platform
**Tail Suspension Test (TST)**: A behavioral test used to assess depressive-like states in mice, based on the duration of immobility when suspended by the tail
**Oxytocin (OXT)**: A neuropeptide and hormone involved in social bonding, stress regulation, and mood modulation; studied here for its potential anxiolytic and antidepressant effects
